# The Relationship between Pulmonary Artery Pressure and Mortality in Type 2 Diabetes: A Fremantle Diabetes Study Phase II and National Echocardiographic Database of Australia Data Linkage Study

**DOI:** 10.3390/jcm12247685

**Published:** 2023-12-14

**Authors:** Nishant Nundlall, David Playford, Geoff Strange, Timothy M. E. Davis, Wendy A. Davis

**Affiliations:** 1 School of Medicine, The University of Notre Dame, Fremantle, WA 6160, Australia; nishantnundlall@gmail.com (N.N.); david@playford.biz (D.P.); gstrange@neda.net.au (G.S.); 2 The Heart Research Institute, Newtown, NSW 2042, Australia; 3 Department of Cardiology, Royal Prince Alfred Hospital, Camperdown, NSW 2050, Australia; 4 Medical School, The University of Western Australia, Fremantle Hospital, Alma Street, Fremantle, WA 6160, Australia; wendy.davis@uwa.edu.au

**Keywords:** type 2 diabetes, pulmonary hypertension, mortality

## Abstract

An elevated estimated right ventricular systolic pressure (eRVSP) identified on echocardiography is present in one-third of individuals with type 2 diabetes, but its prognostic significance is unknown. To assess the relationship between eRVSP and mortality, prospective data from 1732 participants in the Fremantle Diabetes Study Phase II were linked with the National Echocardiographic Database of Australia. Of this cohort, 416 (mean age 70.6 years, 47.4% males) had an eRVSP measured and 381 (91.4%) had previously confirmed type 2 diabetes. Receiver- operating characteristic analysis of the relationship between eRVSP and all-cause mortality was conducted. Survival analyses were performed for participants with type 2 diabetes diagnosed before first measured eRVSP (n = 349). Cox regression identified clinical and echocardiographic associates of all-cause mortality. There were 141 deaths (40.4%) during 2348 person-years (mean ± SD 6.7 ± 4.0 years) of follow-up. In unadjusted Kaplan–Meier analysis, mortality rose with higher eRVSP (log-rank test, *p* < 0.001). In unadjusted pairwise comparisons, eRVSP >30 to 35, >35 to 40, and >40 mmHg had significantly increased mortality compared with eRVSP ≤ 30 mmHg (*p* = 0.025, *p* = 0.001, *p* < 0.001, respectively). There were 50 deaths in 173 individuals (29.1%) with eRVSP ≤ 30 mmHg, and 91 in 177 (51.4%) with eRVSP > 30 mmHg (log-rank test, *p* < 0.001). In adjusted models including age, Aboriginal descent, Charlson Comorbidity Index ≥ 3 and left heart disease, eRVSP > 30 mmHg predicted a two-fold higher all-cause mortality versus ≤ 30 mmHg. An eRVSP > 30 mmHg predicts increased all-cause mortality in type 2 diabetes. Where available, eRVSP could inform type 2 diabetes outcome models.

## 1. Introduction

There is evidence from a number of longitudinal studies conducted in developed countries that diabetes-associated mortality has been decreasing over the past few decades [1,2,3,4,5]. Although this is likely to reflect, at least in part, intensification of cardiovascular disease (CVD) risk factor management [6,7,8], there is still greater mortality in people with versus without type 2 diabetes, especially for all-cause rather than CVD death [9]. A potentially important component of the residual mortality risk in type 2 diabetes could be pulmonary hypertension (PH). PH is a heterogeneous condition that can be associated with significant functional limitation and which can progress to right ventricular failure and premature death [10,11]. It is present when there is a resting mean pulmonary artery pressure of ≥20 mmHg on right heart catheterisation [11]. Although not the definitive investigation for diagnosis of PH [12], Doppler echocardiography can provide a noninvasive estimate of right ventricular systolic pressure (eRSVP). In studies of large general population echocardiographic databases, a mild elevation of eRVSP (>30 mmHg) is a common finding and a robust marker of mortality beyond recognised risk factors such as a reduced left ventricular ejection fraction (LVEF), left heart disease (LHD) with preserved LVEF, increased left atrial volume, and increased left ventricular mass [13,14,15,16].

In people with diabetes, the risk of PH is increased [17,18] and the available evidence suggests that it carries a particularly poor prognosis [19,20]. Whether the presence of an eRVSP >30 mmHg, which was present in approximately one-third of community-based individuals with type 2 diabetes who had undergone echocardiography as part of routine clinical care [21], has the same prognostic significance as in the general population is unknown. The aims of the present study were, therefore, to determine the eRVSP threshold associated with increased mortality in well-characterised type 2 diabetes and to explore its demographic and clinical predictors. 

## 2. Materials and Methods

### 2.1. Study Site, Participants and Approvals

The Fremantle Diabetes Study Phase II (FDS2) is a prospective, observational cohort study of residents with known diabetes (excluding gestational diabetes) recruited from a zip code-defined urban community of 157,000 people in the state of Western Australia (WA). Individuals with a physician-confirmed diagnosis of diabetes were eligible for enrolment. The characteristics of the FDS2 sample, including classification of diabetes and details of nonrecruited participants, have been described previously [22]. Type of diabetes (including previous categorisation by a clinician) was ascertained according to diabetes treatment history, body mass index, age of diagnosis, nature of first presentation, and self-identification. Case records were consulted for evidence of ketonemia and for data on islet autoantibody titres, and serum insulin and C-peptide concentrations. Genetic and serologic screenings were undertaken for maturity onset diabetes of the young and latent autoimmune diabetes of adults (LADA), respectively. The South Metropolitan Area Health Service Human Research Ethics Committee approved FDS2, and written informed consent was obtained from each participant.

### 2.2. Clinical Assessment

Participants in the FDS2 underwent comprehensive face-to-face assessments at baseline and biennially, interspersed with biennial postal questionnaires [22]. At each visit, demographic and clinical information was documented, physical examinations and associated investigations were carried out, and fasting blood and urine samples for biochemical tests were obtained. Ethnic background was categorised according to self-selection, and country of birth and parent/grandparent birthplaces.

### 2.3. Echocardiography Database and Parameters

The National Echocardiographic Database of Australia (NEDA) is an observational registry that captures individual echocardiographic data and basic demographic profiling in a retrospective and prospective basis from participating centres throughout Australia with the capacity to link the data to health outcomes [23]. Reporting conforms to the Strengthening the Reporting of Observational Studies in Epidemiology guidelines [24]. NEDA is registered with the Australian New Zealand Clinical Trials Registry (ACTRN12617001387314). Ethical approval was obtained from all relevant human research ethics committees in each Australian state and territory.

All echocardiographic data are transferred into a central database using a “vendor-agnostic” extraction process that converts every measurement into a standard NEDA format [23]. The eRVSP was derived using the Bernoulli equation (RVSP = TRV² + 5 mmHg), where TRV represents the peak tricuspid regurgitation (TR) velocity and 5 mmHg represents a conservative estimate of the right atrial pressure [15]. Where no TR was present or the TR jet was insufficient to measure a peak velocity, the TRV was assumed to be normal and PH presumed to be absent. The date of the first calculated eRVSP value became the new baseline for the current substudy. LHD was defined as left ventricular ejection fraction <54% (measured using the Simpson biplane method or the Teichholz method if image quality was poor), signs of increased left ventricular ejection filling pressure (ratio of mitral inflow E-wave peak velocity to peak early relaxation tissue Doppler velocity, E/E’ > 12), or left atrial volume index >34 mL/m² and/or hemodynamically significant (greater than mild) mitral or aortic valve disease and previous mitral valve replacement or aortic valve replacement.

### 2.4. Data Linkage

Secure linkage of the NEDA and FDS2 databases was approved by The University of Notre Dame Australia Human Research Ethics Committee (reference 2020-060F). The NEDA database contained 353,093 echocardiograms performed on individuals with FDS2 postcodes and these were matched to FDS2 participants through basic demographic details and, when applicable, date of death. If necessary, height and weight were cross-checked. Where a death was recorded after 2016 through NEDA linkage with the National Death Index (NDI), death dates were validated through the Perth Metropolitan Cemeteries Board database.

### 2.5. Ascertainment of Mortality and Charlson Comorbidity Index

Death data from NEDA and FDS2 were combined. NEDA data are linked to the NDI data held by the Australian Institute for Health and Welfare. Probability matching was used to increase the sensitivity and specificity of mortality data. This linkage provided the vital status of everyone captured by NEDA through advanced probability matching to 23 March 2019. The FDS2 database was linked through the WA Data Linkage System (WADLS) [25] with the WA Registry for Births, Deaths and Marriages to 12 May 2022. The census date for the present study was 23 March 2019. The FDS2 database was also linked through the WADLS with the Hospital Morbidity Data Collection (HMDC) to 31 January 2022. The HMDC data were used to extract the Charlson Comorbidity Index (CCI) [26], excluding codes specific for diabetes and its complications, between 1 January 1980 and the time of the first calculated eRVSP value.

### 2.6. Statistical Analysis

The computer package IBM SPSS Statistics 28 (IBM Corporation, Armonk, NY, USA) was used for statistical analysis. Data are presented as percentages, mean ± SD, geometric mean (SD range), or, in the case of variables that did not conform to a normal or ln-normal distribution, as median and interquartile range [IQR]. Two independent samples were compared using Fisher’s exact test for proportions, Student’s *t*-test for normally distributed variables, and the Mann–Whitney U-test for variables that were not normally or ln-normally distributed. Greater than two independent samples were compared with the Fisher–Freeman–Halton exact test for proportions, ANOVA for normally or ln-normally distributed variables, and the Kruskal–Wallis test otherwise.

Using eRVSP as the continuous variable and all-cause death as the outcome, receiver-operating characteristic (ROC) curves were created. The optimal eRVSP threshold above which mortality increased was based on Youden’s index, which is the value of eRVSP at which the [Sensitivity – (1-Specificity)] is maximised. Survival data were then analysed by eRVSP groups centred on this optimal eRVSP threshold. Baseline for survival analyses was the date of the first measured eRVSP (the earliest date being the 24 August 2001). 

Cumulative survival was compared for each eRVSP category using the Kaplan–Meier analysis (log-rank test). The Cox regression, with time as the time-line and backward conditional stepwise variable selection (entry *p* < 0.050 then manual removal one-by-one of the least significant variables until all *p* < 0.050 in the model) of clinically plausible and available variables with bivariable *p* < 0.20 (except for Aboriginal descent, which was considered for entry regardless due to the much younger age distribution of this group), was used to determine independent baseline predictors of mortality. Echocardiographic variables with more than 10% missing data were not considered for entry into the Cox regression. The proportional hazards assumption was checked using time-varying covariates. 

To determine generalisability of results, age and sex were compared for unmatched versus matched participants, and, for the matched participants, those without and with a calculable eRVSP.

## 3. Results

### 3.1. Participant Characteristics

**Figure 1:**
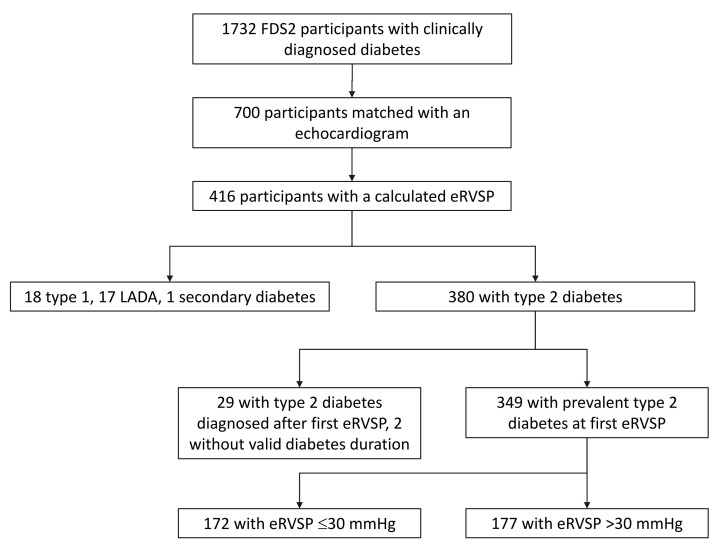
Consort diagram showing selection of Fremantle Diabetes Study Phase 2 participants for inclusion in the present substudy.

Of 4639 eligible residents identified, 1668 (36%) were recruited together with 64 FDS Phase I (FDS1) participants who had moved out of the study area. As shown in Figure 1, 700 participants (40.4% of the total FDS2 cohort) were matched with an echocardiographic study. Compared with the 1032 FDS2 participants who were not matched with an echocardiogram on the NEDA database, the 700 who were matched were significantly older at FDS2 study entry (61.5 ± 14.4 vs. 67.3 ± 11.6 years, *p* < 0.001) but were not significantly more likely to be female (48.7% vs. 46.7%, *p* =0.378). 

Of the 700 validly matched participants, 416 (59.3%) had sufficient TR to calculate an eRVSP either before or after the FDS2 baseline assessment [13]. Compared with the 285 participants who had an echocardiogram but no measurable/measured TR, the 416 with measurable/measured TR were significantly older at the first echocardiogram captured by NEDA (65.0 ± 11.9 vs. 70.1 ± 10.9 years, *p* < 0.001) and were significantly more likely to be female (37.2% vs. 52.8%, *p* < 0.001). Of the 416 individuals with a measurable eRVSP, 380 (91.4%) had type 2 diabetes, 18 (4.3%) type 1 diabetes, 17 (4.1%) LADA, and one (0.2%) had secondary diabetes. Nearly 43% (n = 178) had been hospitalised for/with CHD before, or contemporaneous with, the echocardiographic study at which eRVSP was first calculated. Thirty-one (7.4%) participants had an eRVSP measured before diagnosis of diabetes and two had no valid diabetes duration data. For the remaining 383 participants (349 with type 2 diabetes), the median [IQR] diabetes duration was 14.3 [8.0–20.9] years.

The clinical and echocardiographic characteristics both overall and by eRVSP category are summarised in Table 1. Age and diabetes duration were significantly associated with eRVSP category, as were nonsinus rhythm and LHD. Of the echocardiographic variables with more than 10% missing data, left ventricular end-diastolic diameter (LVEDD), mitral E and A inflow velocities, mitral E/A ratio, septal E/E’, and left atrial (LA) volume index were significantly and positively associated with increasing eRVSP category, in particular, eRVSP >40 mmHg. Echocardiographic characteristics of heart failure with preserved ejection fraction (HFpEF) were typically observed at an eRVSP >40 mmHg.

### 3.2. Estimated Right Ventricular Systolic Pressure All-Cause Mortality Threshold

There were 165 deaths (39.7%) during 2954 person-years (7.1 ± 4.1 years; range 0.01 to 17.6 years) of follow-up. ROC analyses for all-cause mortality by diabetes type are summarised in Table 2. Optimal cut-points were 34 mmHg for type 2 diabetes and 29 mmHg for the small group with type 1 diabetes.

**Figure 2:**
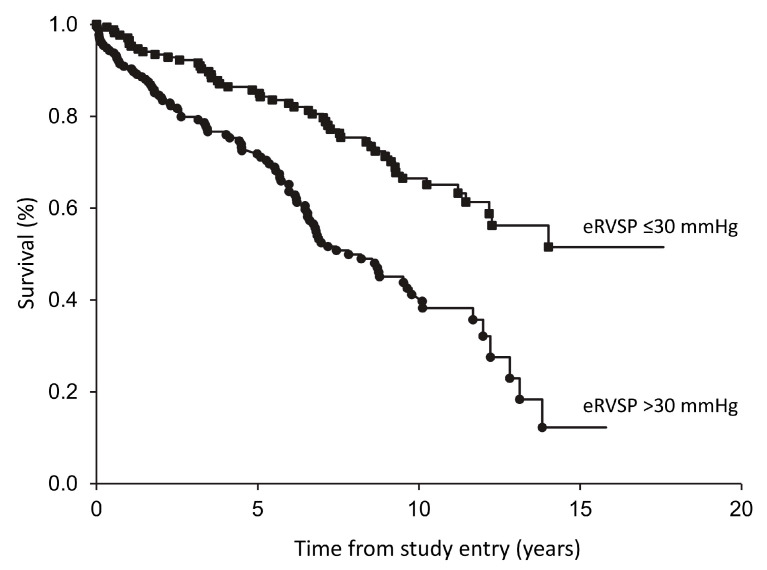
Kaplan–Meier plots of survival in Fremantle Diabetes Study Phase 2 participants categorised by eRVSP.

For homogeneity, only those with prevalent type 2 diabetes at the time of first eRVSP estimation (n = 349) were included in subsequent analyses. For the survival analyses, eRVSP was categorised into four subgroups: ≤30, >30–35, >35–40, and >40 mmHg. There were 141 deaths in these 349 participants (40.4%), including 50 in the 173 (29.1%) with an eRVSP ≤ 30 mmHg, 25 in the 62 (40.3%) with an eRVSP > 30–35 mmHg, 20 in the 37 (54.1%) with an eRVSP > 35–40 mmHg, and 46 in the 78 (59.0%) with eRVSP > 40 mmHg. Kaplan–Meier analysis showed that mortality increased significantly with increasing eRVSP (log-rank test, *p* < 0.001). In unadjusted pairwise comparisons, eRVSP >30–35, >35–40, and >40 mmHg had significantly increased mortality compared with eRVSP ≤ 30 mmHg (*p* = 0.023, *p* = 0.001 and *p* < 0.001, respectively). Those with eRVSP > 30–35 mmHg had significantly lower mortality than >40 mmHg (*p* = 0.018), but no other comparisons were significantly different. Consequently, the three higher categories were combined into one group. Survival was significantly reduced for participants with eRVSP > 30 mmHg with 50 deaths in the 172 individuals (29.1%) with eRVSP ≤ 30 mmHg and 91 deaths in the 177 (51.4%) with eRVSP > 30 mmHg (see Figure 2; log-rank test, *p* < 0.001).

### 3.3. Predictors of Mortality

In bivariable analysis (see Table 3), age, BMI (negatively), diabetes duration, CCI, nonsinus rhythm, LHD, and eRVSP category were significantly associated with all-cause mortality during 2348 person-years (6.7 ± 4.0 years) of follow-up. Of the echocardiographic variables with >10% missing data, those who died during follow-up had significantly higher LV end diastolic diameter, LV mass index, LV ejection fraction, mitral A velocity, septal E/E’, and LA volume index than the survivors.

In backward stepwise Cox regression excluding eRVSP category, age, Aboriginal descent, and CCI ≥ 3, diabetes duration, and LHD were significantly associated with all-cause mortality. After eRVSP was included in the model, each of the three eRVSP categories > 30 mmHg were independently associated with nearly double the mortality risk compared with an eRSVP ≤ 30 mmHg (see Table 4). Combining the three >30 mmHg eRVSP categories, an eRVSP > 30 mmHg independently increased the risk of death nearly two-fold compared with an eRSVP ≤ 30 mmHg (1.92 (1.34, 2.76), *p* < 0.001). The proportional hazards assumption was not violated for any variable in the final model.

## 4. Discussion

This study demonstrated that an eRVSP > 30 mmHg is independently associated with mortality in community-based people with type 2 diabetes. This result is consistent with recent reports that have established the same eRVSP threshold as a predictor of mortality in large general population echocardiographic databases [14,15]. In addition, the detailed FDS2 phenotypic data allowed identification of other independent (and expected) determinants of all-cause death after adjustment for the presence of an elevated RVSP. Increasing age, Aboriginal ethnicity, the presence of LHD, and CCI ≥3 were positively associated with all-cause mortality.

A clear eRVSP mortality threshold of >30 mmHg was previously demonstrated by our group using echocardiographic data that were not specific to diabetes [15], with a mildly raised eRVSP (30.0 to 39.9 mmHg) accounting for more than half of the premature deaths. The present study adds to this knowledge by demonstrating the same eRVSP mortality threshold in diabetes. In the general population, additional echocardiographic features of right ventricular function and morphology are required to increase the probability of correctly identifying PH, with consideration of definitive right heart catheterisation [12]. The present data would suggest that that this recommendation should also be made in the specific case of type 2 diabetes. Whether a mildly raised eRVSP should be seen as an additional diabetes risk-factor is unknown, prompting addition of options such as SGLT-2 inhibitors and/or GLP-1 agonists [27]. However a recent Japanese study has shown an improvement in exercise-induced PH in individuals with type 2 diabetes [28] and preliminary evidence has suggested benefit of these agents in experimental PH [29]. The cause of the observed elevated eRVSP in this cohort is probably multifactorial, and the study was not powered to examine individual contributors to the observed mortality in each eRVSP group. However, typical echocardiographic features of HFpEF were observed in the group with eRVSP > 40 mmHg, where mortality benefits of SGLT-2 inhibitors and GLP-1 agonists have also been observed [30,31].

The eligible participants in the present study represented only a quarter of all FDS2 participants with confirmed type 2 diabetes. In addition, there was restricted availability of data at the time of first valid echocardiograph in this subset of participants as this was mostly not contemporaneous with a regular FDS2 detailed assessment. Nevertheless, the nonechocardiographic independent determinants of death in this subgroup (older age, Aboriginal ethnicity, and high CCI) were in accord with those in the full FDS2 cohort [32], suggesting that the present participants were representative. 

There is a paucity of published data relating to raised RVSP and its consequences in type 1 diabetes. The eRVSP threshold for increased mortality in type 1 diabetes in the present study (>29 mmHg) was very close to that for type 2 diabetes, but larger prospective studies would be needed to confirm this finding.

This study has some limitations. First, only FDS participants who were also identified in NEDA were linked, which raises the possibility of selection bias. However, there were no systematic differences between the linked and unlinked patients including for nonechocardiographic predictors of death, and the >30 mmHg threshold is consistent with other studies. Second, as acknowledged, our sample size was small, comprising 349 participants with confirmed type 2 diabetes and evaluable TR. Third, as also acknowledged, we did not adjust for rich clinical covariates in FDS2 since these were not known at the time of first determination of eRVSP. Fourth, the presence of raised eRVSP was not centrally adjudicated by a core laboratory, and we did not perform right heart catheterisation to verify the presence of PH. Finally, we could not determine whether type 2 diabetes itself was causally related to the eRVSP mortality threshold we identified.

## 5. Conclusions

This is the first study to report a stand-alone echocardiographic eRVSP threshold >30 mmHg as a marker of increased mortality in type 2 diabetes in the community. The clinical importance of this threshold as a potentially modifiable risk factor for all-cause death needs to be explored in other contemporary clinical datasets and with deeper clinical phenotyping. This can potentially lead to better personalisation of diabetes risk stratification and treatment, including through use of portable echocardiography [33] and machine learning-supported interpretation of results [34] that should increase the availability and accuracy of data acquisition.

## Figures and Tables

**Table 1:** Clinical and echocardiographic characteristics overall and by eRVSP category in participants with type 2 diabetes at the time of first valid eRVSP measurement.

	All	≤30 mmHg	>30 to 35 mmHg	>35 to 40 mmHg	>40 mmHg	Trend *p*-Value
Number (%)	349 (100)	172 (49.3)	62 (17.8)	37 (10.6)	78 (22.3)	
Age (years)	71.6 ± 10.3	70.2 ± 10.2	70.5 ± 11.0	73.2 ± 9.4	74.9 ± 9.6 **	0.005
Sex (% male)	47.6	50.0	37.1	35.1	56.4	0.049
APOE4 genotype (%)	22.3	26.2	15.0	16.7	22.1	0.282
Ethnic background (%):						
Anglo–Celt	57.64	59.3	61.3	54.1	52.6	0.671
Southern European	12.6	12.8	8.1	10.8	16.7	0.514
Other European	5.2	6.4	3.2	5.4	3.8	0.778
Asian	4.9	5.2	6.5	2.7	3.8	0.869
Aboriginal	7.2	4.1	8.1	13.5	10.3	0.082
Mixed/other	12.6	12.2	12.9	13.5	12.8	0.995
Overseas born (%)	42.1	45.3	41.9	35.1	38.5	0.601
Not fluent in English (%)	11.5	14.0	8.1	5.4	11.5	0.436
Educated beyond primary level (%)	86.2	89.4	85.0	86.1	79.7	0.238
Age at diabetes diagnosis (years)	57.2 ± 12.3	56.3 ± 12.4	58.3 ± 13.3	58.9 ± 10.9	57.2 ± 12.1	0.580
Diabetes duration (years)	13.6 [7.8–20.2]	12.6 [6.5–20.1]	12.2 [7.1–17.6]	13.5 [8.8–18.3]	18.8 [11.3–22.5] *^,††^	0.007
BMI (kg/m²)	29.7 ± 6.0	29.1 ± 5.9	30.2 ± 5.2	30.9 ± 7.4	29.9 ± 6.3	0.363
CCI (%):					***	<0.001
0	35.0	41.9	38.7	27.0	20.5	
1 or 2	33.8	36.6	32.3	35.1	28.2	
≥3	31.2	21.5	29.0	37.8	51.3	
Nonsinus rhythm ^¶^ (%)	22.3	16.3	19.4	21.6	38.5 **	0.002
LHD (%)	48.1	43.0	38.7	51.4	65.4 *^,††^	0.004
LVDD (cm; n = 248)	4.7 ± 0.6	4.7 ± 0.6	4.7 ± 0.5	4.7 ± 0.7	4.8 ± 0.8	0.381
LV mass index (g/m²; n = 189)	96 (74–123)	94 (74–119)	92 (77–111)	95 (72–126)	110 (84–145) **^,†^	0.002
LVEF (%; n = 271)	63 [57–67]	64 [59–67]	64 [57–69]	64 [55–71]	60 [43–66]	0.064
Mitral E inflow velocity (cm/s; n = 287)	84 (61–116)	74 (57–97)	85 (65–113)*	85 (60–120)	111 (82–150) ***^,†††,‡‡‡^	<0.001
Mitral A inflow velocity (cm/s; n = 271)	76 (51–113)	70 (49–101)	81 (56–117)	80 (49–128)	87 (57–134) **	0.003
Mitral inflow E:A ratio (n = 268)	0.93 (0.63–1.37)	0.87 (0.63–1.19)	0.94 (0.67–1.32)	0.90 (0.63–1.28)	1.18 (0.71–1.98) ***^,†,‡^	<0.001
Septal E/E’ ratio (n = 191)	12.1 (7.8–18.7)	10.9 (7.5–15.8)	10.7 (8.2–14.0)	11.5 (7.3–18.0)	18.8 (11.8–30.2) ***^,†††,‡‡‡^	<0.001
LA volume index (mL/m²; n = 180)	32 (22–47)	30 (21–41)	29 (21–42)	30 (21–44)	44 (30–65) ***^,†††,‡‡^	<0.001

Data are presented as percentages (%), mean ± SD, median [interquartile range, IQR], geometric mean (SD range). ^¶^ Including atrial fibrillation. * *p* < 0.05, ** *p* < 0.01, *** *p* < 0.001 vs. eRVSP ≤ 30 mmHg, ^†^ *p* < 0.05, ^††^ *p* < 0.01, ^†††^ *p* < 0.001 vs. eRVSP > 30 to 35 mmHg, ^‡^ *p* < 0.05, ^‡‡^ *p* < 0.01, ^‡‡‡^ *p* < 0.001 vs. eRVSP >35 to 40 mmHg, adjusted for multiple pairwise comparisons using the Bonferroni correction.

**Table 2:** Youden index for the optimum cutoff for eRVSP for all-cause mortality by type of diabetes.

	All Diabetes Types	Type 2 Diabetes	Type 2 Diabetes with Diabetes Duration ≥0 Years at Time of First Valid eRVSP Measurement	Type 1 Diabetes (All Had Diabetes Duration ≥0 Years at Time of First Valid eRVSP Measurement)
N	416	380	349	18
AUC (95% CI)	0.657 (0.602–0.712)	0.641 (0.583–0.700)	0.646 (0.586–0.707)	0.786 (0.569–1.000)
*p*-value	<0.001	<0.001	<0.001	0.046
Youden’s index	32.14	33.96	33.96	29.02
Sensitivity	0.606	0.544	0.546	0.857
Specificity	0.661	0.706	0.721	0.636

**Table 3:** Clinical and echocardiographic characteristics by all-cause mortality to 29 March 2019 in participants with type 2 diabetes at the time of first valid eRVSP measurement.

	Alive	Deceased	*p*-Value
Number (%)	208 (59.6)	141 (40.4)	
Age (years)	69.6 ± 10.1	74.7 ± 9.8	<0.001
Sex (% male)	46.2	49.6	0.585
APOE4 genotype (%)	20.8	24.6	0.430
Ethnic background (%):			
Anglo–Celt	56.7	58.9	0.741
Southern European	13.5	11.3	0.624
Other European	4.3	6.4	0.462
Asian	5.8	3.5	0.450
Aboriginal	6.3	8.5	0.526
Mixed/other	13.5	11.3	0.624
Born in Australia (%)	43.3	40.4	0.659
Not fluent in English (%)	13.0	9.2	0.308
Educated beyond primary level (%)	86.7	85.4	0.751
Age at diabetes diagnosis (years)	56.4 ± 11.8	58.3 ± 13.0	0.166
Diabetes duration (years)	12.5 [6.6–19.2]	15.6 [10.1–21.5]	0.002
BMI (kg/m²)	30.3 ± 6.0	28.7 ± 5.9	0.023
Charlson Comorbidity Index (%):			<0.001
0	45.7	19.1	
1 or 2	35.6	31.2	
≥3	18.8	49.6	
Nonsinus rhythm ^¶^ (%)	17.3	29.8	0.009
LHD (%)	40.4	59.6	<0.001
eRVSP category (%):			<0.001
≤30 mmHg	58.7	35.5	
>30 to ≤35 mmHg	17.8	17.7	
>35 to ≤40 mmHg	8.2	14.2	
>40 mmHg	15.4	32.6	
LVEDD (cm; n = 249)	4.6 ± 0.6	4.8 ± 0.7	0.029
LV mass index (g/m²; n = 189)	91 (71–117)	105 (83–133)	<0.001
LVEF (%; n = 272)	64 [59–68]	61 [47–66]	0.005
Mitral E inflow velocity (cm/s; n = 288)	82 (61–111)	86 (60–123)	0.252
Mitral A inflow velocity (cm/s; n = 272)	73 (50–107)	81 (54–123)	0.026
Mitral inflow E/A ratio (n = 269)	0.93 (0.66–1.32)	0.93 (0.59–1.47)	0.999
Septal E/E^1^ ratio (n = 191)	11.2 (7.8–16.0)	14.0 (8.2–24.0)	0.004
LA volume index (mL/m²; n = 180)	30 (21–43)	37 (26–54)	<0.001

^¶^ Including atrial fibrillation. Data are presented as percentages (%), mean ± SD, median [interquartile range, IQR].

**Table 4:** Cox regression model for all-cause mortality for participants with type 2 diabetes and diabetes duration ≥ 0.0 years at time of first valid eRVSP measurement.

	Hazard Ratio (95% Confidence Interval)	*p*-Value
Age (increase of 1 year)	1.07 (1.05, 1.09)	<0.001
Aboriginal descent	2.35 (1.17, 4.69)	0.016
CCI ≥ 3	3.66 (2.57, 5.21)	<0.001
LHD	1.93 (1.35, 2.76)	<0.001
eRVSP category:		
<30 mmHg (reference)	1.00	
>30 to ≤35 mmHg	1.86 (1.14, 3.03)	0.013
>35 to ≤40 mmHg	1.89 (1.10, 3.24)	0.021
>40 mmHg	1.98 (1.30, 3.03)	0.002

## Data Availability

Some outcome data supporting the findings of this study are available from the Western Australian Department of Health, but restrictions apply to the availability of these data, which were used under strict conditions of confidentiality for the current study, and so are not publicly available. Data are available from the authors, however, upon reasonable request and with permission of the Western Australian Department of Health.
